# Size Does Matter: Mastectomy Flap Thickness as an Independent Decisional Factor for the Peri-Prosthetic Device Choice in Prepectoral Breast Reconstruction

**DOI:** 10.3390/jcm13237459

**Published:** 2024-12-07

**Authors:** Juste Kaciulyte, Silvia Sordi, Gianluigi Luridiana, Marco Marcasciano, Federico Lo Torto, Enrico Cavalieri, Luca Codolini, Roberto Cuomo, Warren Matthew Rozen, Ishith Seth, Diego Ribuffo, Donato Casella

**Affiliations:** 1 Oncologic Breast Surgery Unit, Department of Medicine, Surgery and Neuroscience, University of Siena, 53100 Siena, Italy; justekc@gmail.com (J.K.);; 2 Unit of Plastic and Reconstructive Surgery, Department of Surgery “P. Valdoni”, Policlinico Umberto I, Sapienza University of Rome, 00185 Roma, Italy; federico.lotorto@uniroma1.it (F.L.T.); diego.ribuffo@uniroma1.it (D.R.); 3 Unit of Oncologic and Breast Surgery, A.R.N.A.S. Brotzu, Businco Oncologic Hospital, 09047 Cagliari, Italy; gianluigi.luridiana@aob.it; 4 Division of Plastic Surgery, Department of Experimental and Clinical Medicine, Magna Graecia University of Catanzaro, 88100 Catanzaro, Italy; 5 Department of Plastic Surgery, Niguarda Ca’ Granda Hospital, 20162 Milano, Italy; 6 Unit of Plastic and Reconstructive Surgery, Department of Medicine, Surgery and Neuroscience, University Hospital of Siena, 53100 Siena, Italy; 7 Department of Plastic and Reconstructive Surgery, Frankston Hospital, Peninsula Health, Melbourne, VIC 3199, Australia

**Keywords:** implant-based breast reconstruction, prepectoral breast reconstruction, acellular dermal matrix, synthetic mesh, cadaver-derived dermal graft

## Abstract

**Background.** In alloplastic breast reconstruction, the choice of implant positioning and the selection of periprosthetic devices is a critical and challenging decision. Surgeons must navigate between various biologic and synthetic meshes, including acellular dermal matrices (ADM). This study aimed to propose a simple selection tool for periprosthetic devices in prepectoral breast reconstruction. **Methods.** Patients scheduled for mastectomy followed by implant-based breast reconstruction between September 2019 and December 2023 were included. Preoperative risk assessments were performed using the Pre-Bra Score, and only those deemed suitable for prepectoral implant placement were selected. Mastectomy flap thickness was used as an independent criterion, and only cases with flap thicknesses less than 1 cm were included. **Results.** A total of 70 cases with an average flap thickness of 0.7 cm (range, 0.4–0.9 cm), as measured by preoperative contrast-enhanced spectral mammography (CESM), underwent prepectoral reconstruction with ADM covering the implant. Of these, 25 patients (35%) received direct-to-implant reconstruction, while 45 (65%) underwent two-stage reconstruction with a temporary tissue expander. Postoperative complications were recorded during a minimum follow-up period of 6 months. Over an average follow-up duration of 17.5 months (range 6–36 months), no major complications were observed. Minor complications occurred in seven patients: infection (1.28%), seroma (3.85%), and superficial skin necrosis (1.28%). Additionally, 21 patients (30%) experienced rippling, and secondary lipofilling was scheduled. **Conclusions.** The incidence of rippling was reduced by 40% through ADM in this patient subgroup, reducing the need for secondary aesthetic refinements.

## 1. Introduction

Prepectoral implant-based breast reconstruction was first introduced in the 1960s, coinciding with the development of silicone gel and saline-filled implants, marking the beginning of modern breast reconstruction [1,2,3]. Following a decline in popularity during the 1970s, this technique experienced a resurgence in 2007 to avoid animation deformity and the pain associated with submuscular reconstructions. This revival occurred alongside advancements in diagnostic, therapeutic, and surgical methods, including skin-sparing mastectomies (SSM) and nipple-sparing mastectomies (NSM), which are collectively referred to as “conservative mastectomies”. These procedures are oncologically safe and feasible and are now considered the gold standard in radical breast surgeries [1,2,3,4]. Both SSM and NSM often result in a mismatch between the overlying skin envelope and the smaller submuscular pocket, further driving the demand for prepectoral reconstruction.

The use of acellular dermal matrix (ADM) in breast reconstruction initially aimed to enhance the volumetric capacity of the submuscular pocket, which eventually led to the logical next step: the preservation of the pectoralis muscle by wrapping the subcutaneous implant in ADM [5,6,7]. Prepectoral breast reconstruction using ADM has demonstrated significant advantages, including reductions in postoperative pain, capsular contracture, and animation deformity. These benefits have led to sustained interest in the technique and the development of alternative devices. However, ADMs have also been associated with increased risks of seroma formation, periprosthetic infection, and Red Breast syndrome. Consequently, surgeons began exploring the use of synthetic meshes as an alternative. In 2014, Casella et al. [8] demonstrated the safety and feasibility of prepectoral implant placement using a non-resorbable titanium-coated polypropylene synthetic mesh (TiLoop). Subsequently, a variety of absorbable (e.g., Vicryl) and long-term absorbable (e.g., T.I.G.R. Phasix or P.D.S. Durasorb) meshes were developed [9,10].

One notable complication associated with using subcutaneous synthetic meshes, particularly in cases with thinner mastectomy flaps, is the occurrence of rippling. In response, secondary refinement procedures, such as autologous fat grafting, have yielded promising results in addressing this aesthetic issue and expanding the indications for prepectoral breast reconstruction to patients with lower body mass index (BMI) [11].

Today, biologic and synthetic meshes have demonstrated success in prepectoral breast reconstruction by providing improved pocket control, reliable implant coverage, and reduced rates of capsular contracture [12]. The optimal implant placement and the periprosthetic device are crucial for successful outcomes. In 2021, our team introduced the Pre-Bra Score, a risk assessment tool designed to guide surgeons in selecting the most suitable implant-based breast reconstruction technique for each patient [13]. Since then, we have expanded our research by systematically applying the Pre-Bra Score in clinical practice and developing an application that facilitates its usage. Given the wide range of available ADM and synthetic mesh products, a comprehensive understanding of their appropriate applications is essential. This preliminary study aims to describe a straightforward selection tool for surgeons practising prepectoral breast reconstruction, aiding in the choice of periprosthetic devices.

## 2. Methodology

From September 2019 to December 2023, patients scheduled for skin-sparing mastectomy or nipple-sparing mastectomy were enrolled at the Breast Unit of Azienda Ospedaliera Universitaria Senese in Siena, Italy. The inclusion criteria included patients aged over 18 with a confirmed diagnosis of breast cancer or a genetic predisposition, who were willing and eligible for implant-based immediate breast reconstruction (Table 1). All patients underwent preoperative risk assessment using the Pre-Bra Score. Those with a low score (0–4), indicating a high risk for prepectoral reconstruction, were excluded from the current series and underwent submuscular implant placement with pectoral muscle denervation. The prepectoral breast reconstruction technique, using either a definitive prosthesis or a temporary tissue expander with synthetic mesh, has been extensively described in our previous publications [14]. Briefly, following glandular excision, mastectomy flaps were evaluated for thickness and viability. Reconstruction was performed using either direct-to-implant (DTI) or a two-stage tissue expander approach based on the definitive risk assessment score. Mentor (Mentor Worldwide, Santa Barbara, USA) provided all implants used in this study.

Flap thickness was assessed intra-operatively with a sterile surgical ruler and used as an independent variable for selecting the most appropriate periprosthetic device. In cases where the mastectomy flaps had intermediate or high thickness (between 1 and 2 cm, and greater than 2 cm), implants were wrapped in a titanium-coated polypropylene synthetic mesh (TiLoop Bra, pfm medical, Cologne, Germany). In our experience, when flap thickness exceeds 2 cm, the performance differences between ADM and synthetic mesh become negligible, and the choice of material is left to the surgeon’s preference.

For mastectomy flaps thinner than 1 cm, prepectoral reconstruction was performed by covering the anterior surface of the implant with an acellular dermal matrix, specifically using DED, a human cadaver-derived dermal graft (hADM) provided by the Regional Skin Bank of Siena. Within the European Union, hADMs are processed and released by official tissue establishments, such as skin banks, and can only be obtained through donation. The application of hADMs in wound healing and breast surgery has been well-documented [15].

**Figure 1:**
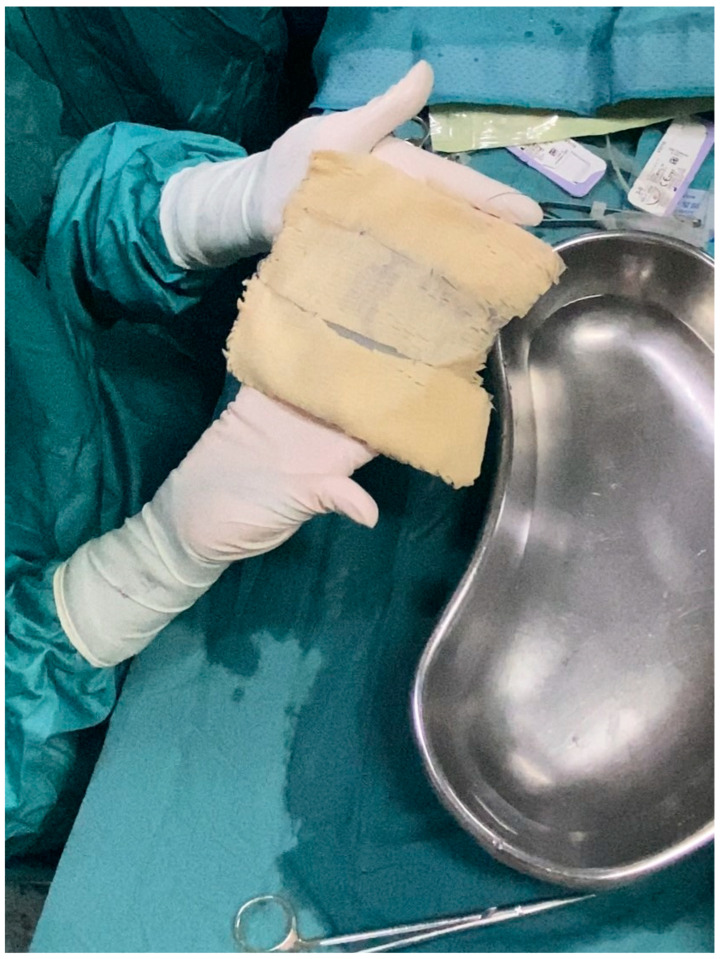
Intraoperative picture of a tissue expander covered with the made-to-measure ADM, ready to be inserted into the mastectomy pocket. The ADM (DED) shields were soaked into an antibiotic solution for 20 min before being secured to each other with single Vicryl 3/0 stitches and to the resorbable synthetic mesh with continuous Vicryl 3/0 suture.

**Figure 2:**
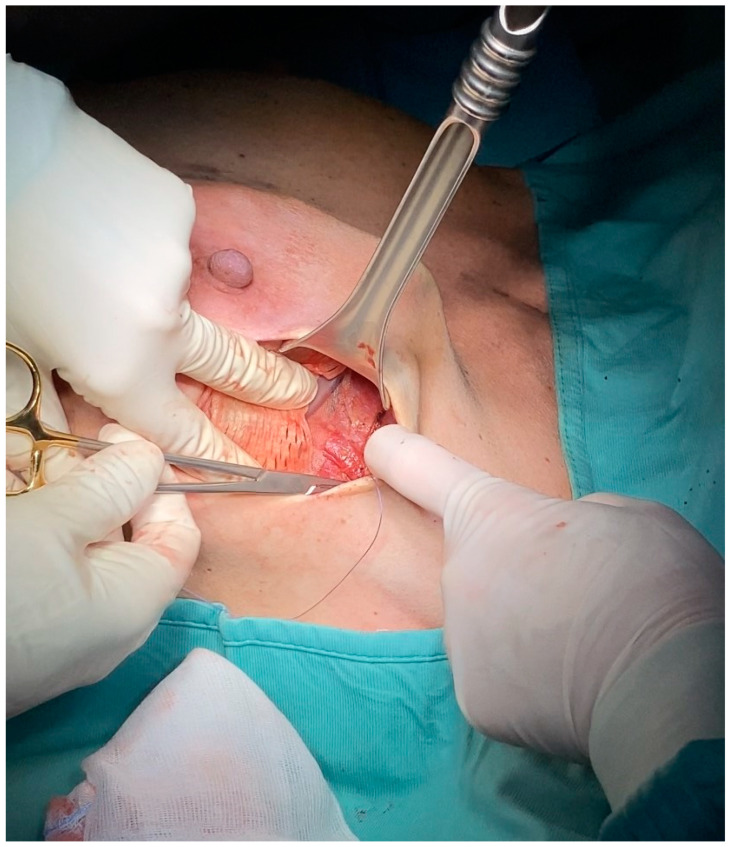
Intraoperative picture of prepectoral breast reconstruction with tissue expander covered with the custom-made ADM device. Once inserted, the implant is secured to the pectoralis major muscle fascia with absorbable stitches on the ADM’s apical, medial, and lateral borders.

In the current study, posterior implant coverage was completed with an absorbable synthetic Vicryl mesh (Ethicon, Raritan NJ, USA), which assisted in distributing tension forces while avoiding direct contact with the overlying mastectomy skin flaps (Figure 1). The anterior hADM and the posterior Vicryl mesh were sutured together with continuous resorbable Vicryl sutures caudally and laterally to create a pocket for the implant. The devices were secured to the pectoral fascia using absorbable sutures along the apical, medial, and lateral borders (Figure 2 and Video S1). A single vacuum drain was placed until the daily fluid output was reduced to approximately 30 cc, and patients were administered oral antibiotics until drain removal. For two-stage reconstructions, the tissue expander was intraoperatively filled to 60% of its capacity, with subsequent expansions starting three days after surgery and repeated once or twice every 15 days. The average time to replace the tissue expander with the definitive implant was six months.

**Figure 3:**
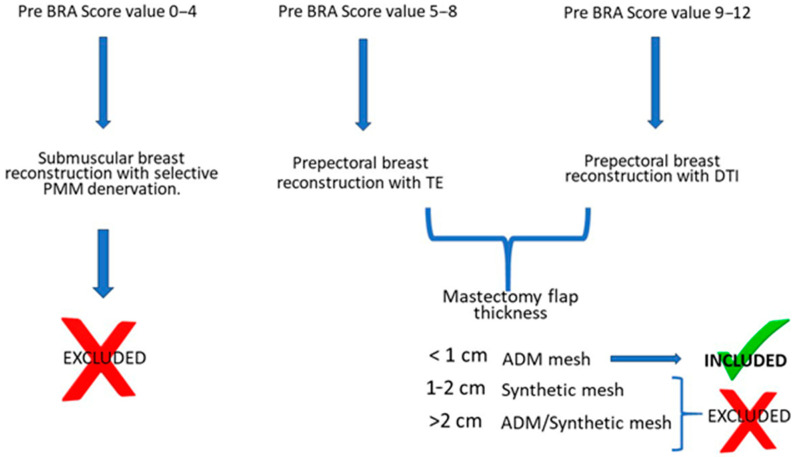
Decisional algorithm applied in the current study to select patients for prepectoral reconstruction (Pre-Bra Score ≥ 5) with a definitive prosthesis (Pre-Bra Score ≥ 9) or a tissue expander (Pre-Bra Score: 5–8) covered with biologic mesh (mastectomy flap thickness < 1 cm).

Eligibility for prepectoral reconstruction (Pre-Bra Score ≥ 5) and the indication for biologic mesh coverage (mastectomy flap thickness < 1 cm) were the final inclusion criteria for this study (Figure 3). Borderline cases with a Pre-Bra Score of 5 to 9 underwent intraoperative indocyanine green (ICG)-based fluorescent angiography to assess flap perfusion.

The minimum follow-up period was six months, with visits scheduled at one month, three months, six months, and annually after that. Postoperative complications and capsular contracture grades were recorded during follow-up. At the six-month follow-up, an expert panel of three plastic surgeons, none of whom performed the surgeries, evaluated the incidence of the rippling phenomenon. Rippling was only recorded in cases where secondary fat grafting was required.

## 3. Results

**Figure 4:**
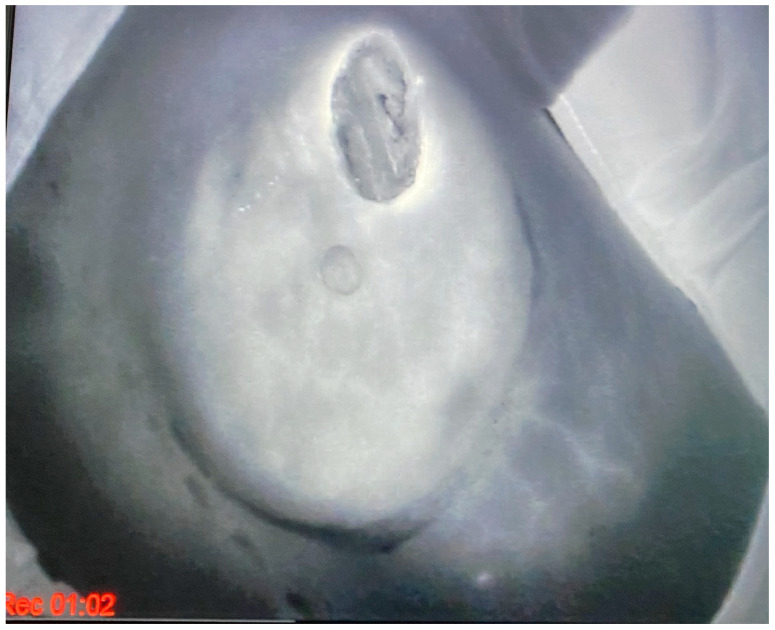
Intraoperative mastectomy flaps check with indocyanine green (ICG)–based fluorescent angiography that shows whole tissues’ vitality even if flaps’ thickness is <1 cm.

Between 2019 and 2023, 70 patients undergoing skin-sparing mastectomy or nipple-sparing mastectomy with immediate prepectoral reconstruction and biologic mesh implant coverage were included in this study. Table 2 summarizes their baseline characteristics, while Table 3 lists the surgical procedures performed. The average age of patients was 51.1 years (range: 32–72 years), with a mean body mass index of 22.7 kg/m^2^ (range: 19–26 kg/m^2^). One patient (1.43%) had diabetes, and five (7.4%) reported smoking. Six patients (8.6%) were BRCA1/2 mutation carriers. The average Pre-Bra Score was 8 (range: 5–10). Of the patients, 35% had a low-risk profile and underwent direct-to-implant procedures (25 cases), while the remaining 65% (45 cases) received two-stage reconstruction with a temporary tissue expander. Intraoperative indocyanine green-based angiography confirmed the vitality of mastectomy flaps in all cases (Figure 4). Flap thickness ranged from 0.4 to 0.9 cm, with an average thickness of 0.7 cm. A total of 78 mastectomies were performed, including 8 (11.43%) bilateral cases. Drains were typically removed after an average of 7 postoperative days (range: 4–11 days).

**Figure 5:**
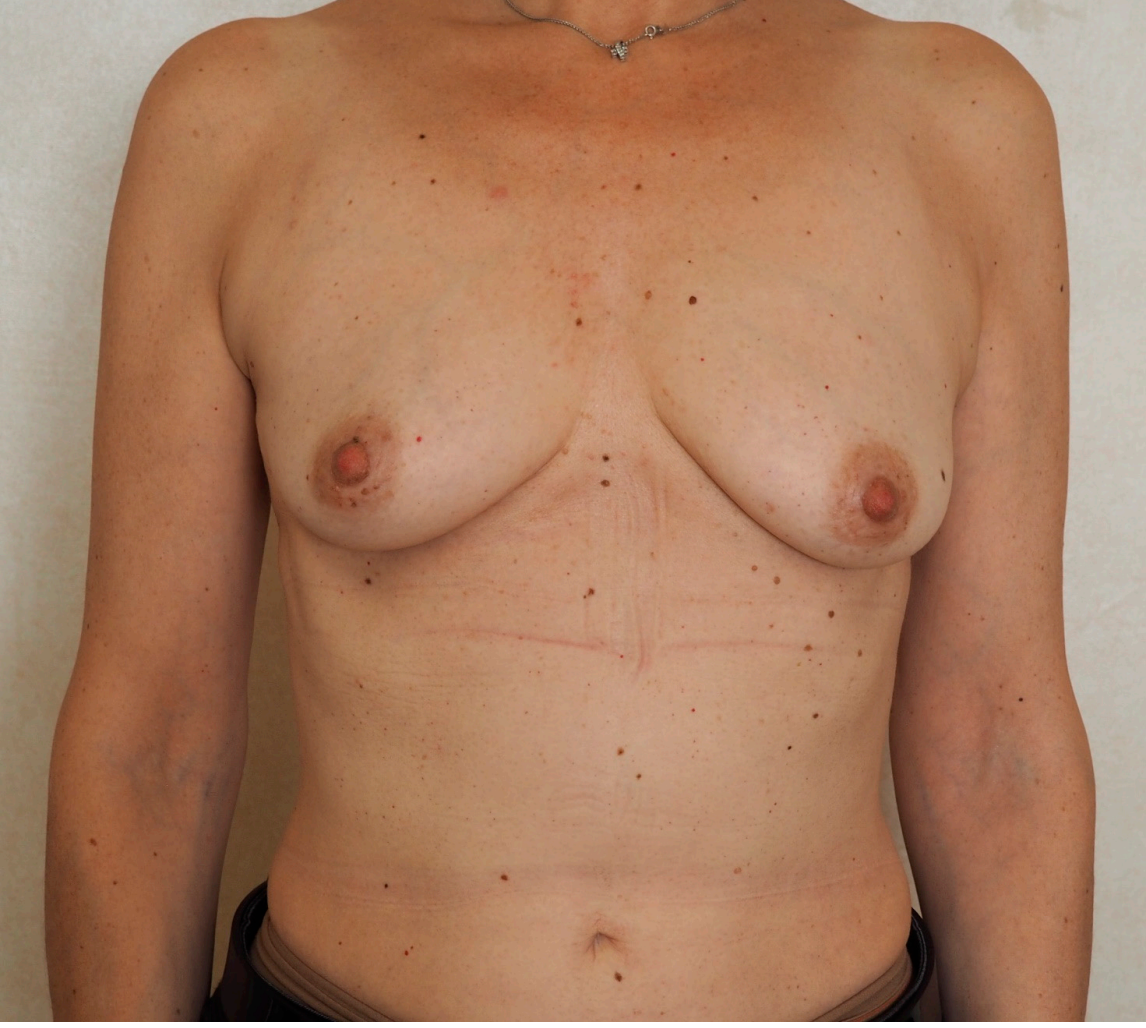
Preoperative picture of a 32-year-old patient (Case 1) who is a BRCA1 mutation carrier and was scheduled for prophylactic bilateral mastectomy. Preoperative risk assessment score for breast reconstruction was high (Pre-Bra Score: 10) and prepectoral DTI reconstruction was planned.

**Figure 6:**
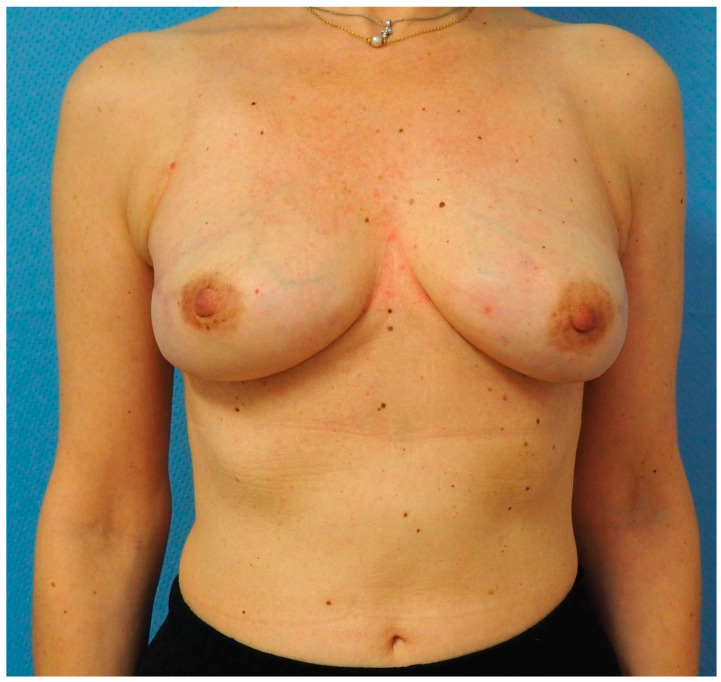
Postoperative picture of Case 1 patient, 6 months after bilateral nipple-sparing mastectomy. Given the high Pre-Bra Score result (10), prepectoral DTI reconstruction was feasible, even if mastectomy flaps resulted <1 cm thick. The subcutaneous implants were covered with ADM. No postoperative complication was registered. At the follow-up visit, the patient presented no capsular contracture and no signs of rippling.

**Figure 7:**
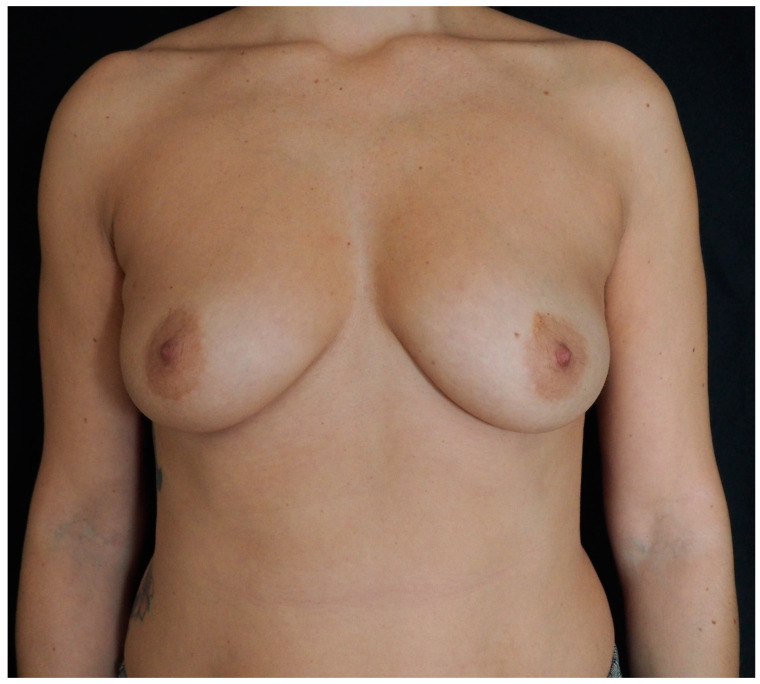
Preoperative picture of a 39-year-old patient (Case 2) with a left breast cancer diagnosis who was scheduled for nipple-sparing mastectomy and sentinel lymph node biopsy. The preoperative risk assessment score for breast reconstruction resulted in medium (Pre-Bra Score: 8), and prepectoral reconstruction with tissue expander was planned.

**Figure 8:**
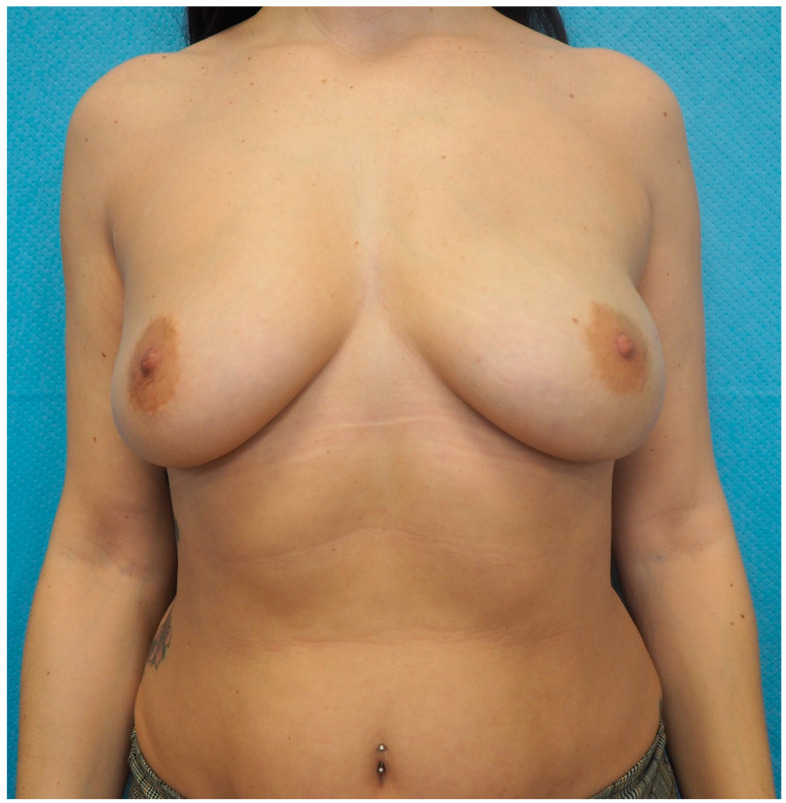
Postoperative picture of Case 2 patient, 8 months after second reconstructive surgery with the definitive implant in place. Given the medium Pre-Bra Score result (8) and the thin mastectomy flaps (<1 cm) assessment, prepectoral reconstruction with tissue expander covered with ADM was performed. No postoperative complications nor rippling were registered.

During a follow-up period ranging from 6 to 36 months (mean: 17.5 months), no major postoperative complications necessitating re-operation were recorded (Figure 5, Figure 6, Figure 7 and Figure 8). Minor early complications occurred in 7 cases (8.97%): 1 patient (1.28%) developed an infection, 3 patients (3.85%) experienced seroma (peri-prosthetic serous fluid collection that required fine needle aspiration during wound dressing changes), and 1 patient (1.28%) exhibited superficial skin necrosis. Early complications were defined if they occurred within 30 days of operation and late ones were classified after that period. All of these cases were managed successfully with wound dressings and oral antibiotics in the outpatient setting (Table 4). Significant capsular contracture (Baker grade III–IV) was observed in only 1.43% (1 patient).

In this cohort, 21 patients (30%) developed rippling in the upper-inner quadrants (UIQ) and were scheduled for secondary corrective fat grafting. Approximately 50 mL of lipofilling per case effectively reduced the visibility and palpability of the implants. A patient’s satisfaction with the reconstruction appearance and no request for further revision surgery was considered as rippling correction achievement. The incidence of secondary lipofilling in these patients was compared to a retrospective control group, consisting of patients with similar risk profiles and thin mastectomy flaps who had undergone subcutaneous implant reconstruction using synthetic mesh (TiLoop). In this control group, 70% of cases required secondary lipofilling due to rippling, highlighting a significant reduction in rippling in the biologic mesh group.

## 4. Discussion

The prepectoral breast reconstruction technique, being less invasive and technically simpler, aligns with the fundamental principle of the plastic surgery reconstructive ladder. The placement of implants in a subcutaneous plane enables shorter operative times, reduces postoperative pain, and decreases the risk of functional impairments in the upper arm and shoulder, particularly when compared to dual-plane reconstructions [16]. With the prepectoral approach’s rise, biologic and synthetic meshes have gained prominence as periprosthetic devices. These materials offer improved control over implant pocket shape and favourable aesthetic results. However, early reports of higher postop-erative complications, such as seroma and infection, limited their widespread use [17]. Briefly, ADMs are soft, de-cellularised tissue grafts composed of extracellular matrix. They act as a scaffold for the patient’s cells to grow, creating an extra tissue layer [18]. ADMs have various shapes, textures and origins: porcine- and bovine-derived ones are widespread in the world, while the commercialised human-derived ones are not contemplated by the European legislations, to date. As an exception, ADMs derived from human derma and processed with complete donor cell removal can be provided by Tissue Establishments as dermal allografts, when authorised by Competent Authorities.

On the other side, meshes are knitted from permanent or absorbable synthetic fibres and act as an internal bra, supporting the implant while integrating into the surrounding tissues [19].

Nowadays, surgeons are investigating the best way to apply these extremely useful yet not thoroughly understood devices. In this regard, Chopra et al. [20] published a comprehensive review over the use of ADMs and synthetic meshes in prepectoral breast reconstruction, showing results comparable to submuscular reconstructions, with good cosmesis and low morbidity. In 2021 Whisker et al. [21] published the joint guidelines from the Association of Breast Surgery and the British Association of Plastic, Reconstructive and Aesthetic Surgeons. Their paper collected recommendations concerning ADMs and synthetic mesh management in breast reconstruction, in particular about their characteristics to consider together with the patient before surgery. Yet, no guidelines nor recommendations have been given to choose which peri-prosthetic device to use to date.

In 2014, our unit introduced the use of non-absorbable titanium-coated polypropylene meshes to wrap the implant in prepectoral breast reconstructions [22]. The outcomes reported were achieved partially to the synthetic meshes’ properties, as the titanised surface allows good cell growth and lower levels of scarring, shrinkage, and inflammation. Moreover, like other meshes, they are known to reduce capsular contracture rate and provide a low inflammatory response [23]. The accurate selection of a targeted reconstructive technique for each patient with the Pre-Bra Score ^13^ helped us to achieve outcomes in line with the literature, as well as our standard operative process that contemplates the same surgeon, oncologic or plastic, performing both the demolitive and reconstructive parts. It has been reported that a dual-trained surgeon performing the whole procedure is associated with improved patient care and breast reconstruction rates compared to the traditional dichotomic approach [24]. We identify with this philosophy in our multidisciplinary group, where mutual skills are shared and consolidated with good results.

As there is no ideal surgery for all patients, no perfect peri-prosthetic device suits all cases. Despite their benefits, synthetic meshes come with widespread concerns, such as fears of foreign body reactions that may lead to inflammation and tissue erosion [25]. In contrast to these common opinions, several studies compared the outcomes of submuscular and prepectoral reconstructions with ADMs and synthetic meshes, failing to show the superiority of any device [26,27,28]. In this regard, Makarewicz et al. [29] carried out a systematic review to create the first comprehensive evaluation of the pros and cons of the two kinds of devices. They found that synthetic meshes appear at least equivalent to ADMs, thus suggesting prioritising their use when possible. In contrast, the paper published by D. Gschwantler-Kaulich et al. [30] indicates the use of ADM for thin skin coverage under 8 mm, in revisional breast surgery or after irradiation where the TiLoop meshes appear not recommended.

With this variety of peri-prosthetic devices at our disposal, a clear and comprehensive identification of their proper applications appears mandatory. In order to face this chaotic scenario, we applied our experience with the Pre-Bra assessment score to select which biologic or synthetic mesh to use in our prepectoral breast reconstructions. As in the selection protocol for the most proper breast reconstructive procedure, we did not identify absolute indicating or contraindicating factors when choosing the peri-prosthetic device. Previous breast surgeries or oncologic therapies did not impede the use of synthetic mesh in our antecedent prepectoral reconstruction experience, showing once again that singular risk factors cannot be considered decisive, nor can the so-dreaded radiotherapy. Thinner mastectomy flaps did not represent an absolute contraindication, even if a consistent number of secondary lipo filling procedures was registered to face the rippling phenomenon.

In the current study, patients who scored high or medium results with the Pre-Bra system were elected for prepectoral implant placement. Patients with the highest score (9–12) received DTI prepectoral reconstruction, while the moderate risk cases (Pre-Bra Score: 5–8) underwent the subcutaneous tissue expander positioning first. All these cases underwent intraoperative evaluation of mastectomy skin flaps vitality and thickness in order to confirm the prepectoral reconstruction indication and to select the device to cover the implant. Those that presented thick and intermedium thickness mastectomy flaps (>2 cm and 1–2 cm) received our previously described titanium-coated polypropylene mesh envelope, while the cases with thinner flaps (<1 cm) were subjected to implant coverage with the biologic ADM meshed graft.

The subcutaneous implant coverage with biologic tissue in the selected cases allowed us to achieve a statistically significant reduction in the necessity for secondary lipofilling for rippling. Indeed, we estimated that up to 60% of our overall patients with prepectoral breast reconstruction undergo secondary lipofilling. For a retrospective comparison, our data were revised since the start of Pre-Bra Score use in 2020. Patients selected for prepectoral reconstruction (intermedium and high score) but with intraoperative assessment of mastectomy flaps thinner than 1 cm represented a subgroup that necessitated secondary lipofilling in 70% of cases. According to the current study, patients presenting the same risk assessment lowered their necessity for lipofilling to 40% of cases. In our experience, the use of subcutaneous synthetic meshes increases the risk of rippling appearance in patients with thin mastectomy flaps. In particular, the UIQ is the area with major incidence of rippling. UIQ is also known as the “no man’s land” [31], as post-surgical defects in this area tend to create more patient dissatisfaction due to its anatomy, with scarce subdermal fat if compared to the rest of breast tissues. By switching to a biologic mesh, we aimed to reduce the incidence of rippling in these selected cases and the necessity for secondary aesthetic refinements such as lipofilling. Indeed, biologic meshes have been associated with low capsular contracture rates and inflammatory response, suggesting a rapid and efficient integration into human tissues [32,33]. These characteristics may explain the lower rippling incidence in the specifically selected patients to which it was applied. Moreover, obtaining the device from the cadaver bank of our hospital allowed us to avoid the higher costs usually associated with biologic meshes.

This study offers several strengths, including the development and systematic use of the Pre-Bra Score to tailor the selection of periprosthetic devices in prepectoral breast reconstruction. By focusing on mastectomy flap thickness as an independent decisional factor, the study provides a clear, objective criterion to guide the choice between biologic and synthetic meshes. The study also highlights the role of biologic meshes, such as ADMs, in reducing rippling in cases with thin mastectomy flaps, contributing valuable insights to a challenging clinical problem. The detailed follow-up and comparison with a retrospective control group further support the reliability of the findings, particularly in demonstrating a reduction in secondary lipofilling procedures when biologic mesh is used.

However, this study has notable limitations. The retrospective design introduces potential biases and limits the ability to establish causality between the interventions and outcomes. Additionally, the sample size is relatively small, and the follow-up period, although sufficient for early complication assessment, may not capture long-term outcomes such as capsular contracture or aesthetic satisfaction over time. Even more, the lack of a control group for the assessment of the main outcomes represents an additional major drawback. Another limitation is the availability of biologic meshes, as the study was dependent on the limited supply from a tissue bank, which could affect the consistency of device selection. Moreover, the use of different types of biologic meshes (DED and bovine pericardium) in some cases could introduce variability in the results, making direct comparisons more challenging. Future studies with larger, multicentre cohorts and prospective designs are necessary to validate these findings and provide more definitive guidance on device selection. One more boundary met during the study was the disposal of DED that is limited to the quantities of product available in the tissue bank at the surgery. In cases where ADM was requested but no DED was available, an alternative biologic mesh made of bilayered bovine pericardium and proven to be as safe as other ADMs yet with lower costs [34,35] was used (Exaflex-MAGGI Srl, Torino, Italy).

## 5. Conclusions

Since its resurgence, prepectoral breast reconstruction has expanded the possibilities in breast surgery by introducing various devices to enhance surgical outcomes. With a wide range of available tools, accurate patient selection is essential to determine the most appropriate device for each case. Building on our previous experience in developing the Pre-Bra Score for selecting patient-tailored reconstructive procedures, we have refined this assessment tool to assist surgeons in choosing the optimal periprosthetic device for prepectoral reconstructions. Given the lack of consensus on the superiority of acellular dermal matrices versus synthetic meshes, this study seeks to clarify the issue, emphasising the importance of customising breast reconstruction to each patient, down to the smallest surgical detail. Our preliminary findings suggest that ADMs can effectively reduce the incidence of rippling in cases with thin mastectomy flaps, providing a strong indication for their use in such scenarios.

## Figures and Tables

**Table 1:** Inclusion criteria to select patients for the current study.

Inclusion Criteria
Age > 18 Years
Breast cancer diagnosis or BRCA1-2 mutation carriers
PreBra Score ≥ 5
Flap thickness < 1 cm

**Table 2:** Baseline patients’ characteristics.

Characteristics	Value (%)
No. of patients	70
Age, yr	
Mean	55
Range	32–72
BMI kg/m^2^	
Mean	23
Range	19–26
Smoking	
Active smokers	5 (7)
Ex-smokers	15 (21)
Never smokers	50 (71)
Diabetes	1 (1)
Previous Radiotherapy	4 (6)
Previous Breast Surgery	12 (17)
BRCA 1-2 mutation carriers	6 (9)
Pre-Bra Score mean	8

Abbreviation: BMI, Body Mass Index.

**Table 3:** Surgical procedures performed on the study’s patients.

Characteristics	Value (%)
Total number of mastectomies	78
monolateral	62 (89)
bilateral	8 (11)
Type of prepectoral breast reconstruction	
Two-stage with TE	49 (63)
DTI	29 (37)
Axillary surgery	
None	6 (8)
SBN	71 (91)
AD	1 (1)

Abbreviations: TE, Tissue Expander. DTI, Direct to Implant. SNB, Sentinel Node Biopsy. AD, Axillary Dissection.

**Table 4:** Early and late complications of included patients.

	Value (%)
Early Complication	7 (9)
Seroma	3 (4)
Superficial skin or nipple necrosis	1 (1)
Haematoma	2 (3)
Infection	1 (1)
Late complication	22 (28%)
Rippling	21 (30)
Severe CC (Beker III–IV)	1 (1)

Abbreviations: CC, Capsular Contracture.

## Data Availability

The data that support the findings of this study are available on request from the corresponding author.

## Supplementary Materials

The following supporting information can be downloaded at: https://www.mdpi.com/article/10.3390/jcm13237459/s1, Video S1: The video shows intraoperative preparing of the made-to-measure peri-prosthetic device for prepectoral breast reconstruction in selected cases with thin (<1 cm) mastectomy flaps. The custom-made device’s frontal wall is created by suturing 3 shields of ADM (DED) together. The posterior wall consists of synthetic resorbable Vicryl mesh secured to the ADM caudally and laterally with continuous resorbable Vicryl sutures. All the suture knots were placed in the inner face of the device. Once the tissue expander is ready (deflated and filled with saline solution up to 1/3 of its definitive volume), it is inserted into the ADM coverage, than placed in the mastectomy prepectoral pocket and secured to the pectoral fascia with absorbable stitches on its apical, medial and lateral borders.
